# Using a Quasi-Experimental Study to Examine Outcomes for Older Adult Intergenerational Program Participants

**DOI:** 10.1093/geroni/igaa057.1767

**Published:** 2020-12-16

**Authors:** Skye Leedahl, Melanie Brasher, Erica Estus

**Affiliations:** University of Rhode Island, Kingston, Rhode Island, United States

## Abstract

To more rigorously examine the University of Rhode Island Cyber-Seniors Program, we conducted a quasi-experimental study to examine if older adult senior center participants (n=25) improved scores on social and technological measures compared to a sample of senior center participants (n=25) who did not take part in the program. Findings showed that participants improved on technology measures compared to the non-participants, including searching and finding information about goods & services, obtaining information from public authorities or services, seeking health information, sending or receiving emails, and participating in online social networks (p<.05). However, participants did not change on social measures. There is either a need to identify better social measures to understand the social benefits of taking part, or to bolster the program to aid in helping older adults alleviate isolation and loneliness. Information on best practices and challenges for gathering outcomes from older participants will be discussed. Part of a symposium sponsored by Intergenerational Learning, Research, and Community Engagement Interest Group.

